# The importance of basal-temporal white matter to pre- and post-surgical naming ability in temporal lobe epilepsy

**DOI:** 10.1016/j.nicl.2022.102963

**Published:** 2022-02-09

**Authors:** Erik Kaestner, Alena Stasenko, Sharona Ben-Haim, Jerry Shih, Brianna M. Paul, Carrie R. McDonald

**Affiliations:** a Center for Multimodal Imaging and Genetics, University of California, San Diego, CA, USA; b Department of Psychiatry, University of California, San Diego, CA, USA; c Department of Neurosurgery, University of California, San Diego, CA, USA; d Department of Neurology, University of California –San Francisco, San Francisco, CA, USA; e San Diego State University, University of California San Diego Joint Doctoral Program in Clinical Psychology, San Diego, CA, USA

## Abstract

**Objective:**

Emerging research highlights the importance of basal-temporal cortex, centered on the fusiform gyrus, to both pre-surgical naming ability and post-surgical naming outcomes in temporal lobe epilepsy (TLE). In this study, we investigate whether integrity of the white matter network that interconnects this basal region to the distributed language network affects naming ability and risk for post-surgical naming decline.

**Methods:**

Patients with drug-resistant TLE were recruited from two epilepsy centers in a prospective longitudinal study. The pre-surgical dataset included 50 healthy controls, 47 left TLE (L-TLE), and 41 right TLE (R-TLE) patients. All participants completed pre-surgical T1- and diffusion-weighted MRI (dMRI), as well as neuropsychological tests of auditory and visual naming. Nineteen L-TLE and 18 R-TLE patients underwent anterior temporal lobectomy (ATL) and also completed post-surgical neuropsychological testing. Pre-surgical fractional anisotropy (FA) of the white matter directly beneath the fusiform neocortex (i.e., superficial white matter; SWM) and of deep white matter tracts with connections to the basal-temporal cortex [inferior longitudinal fasciculus (ILF) and inferior frontal occipital fasciculus (IFOF)] was calculated. Clinical variables, hippocampal volume, and FA of each white matter tract or region were examined in linear regressions with naming scores, or *change* in naming scores, as the primary outcomes.

**Results:**

Pre-surgically, higher FA in the bilateral ILF, bilateral IFOF, and left fusiform SWM was associated with better visual and auditory naming scores (all *p*s < 0.05 with FDR correction). In L-TLE, higher pre-surgical FA was also associated with less naming decline post-surgically, but results varied across tracts. When including only patients with typical language dominance, only integrity of the right fusiform SWM was associated with less visual naming decline (*p* = .0018).

**Discussion:**

Although a broad network of white matter network matter may contribute to naming ability pre-surgically, the reserve capacity of the contralateral (right) fusiform SWM may be important for mitigating visual naming decline following ATL in L-TLE. This shows that the study of the structural network interconnecting the basal-temporal region to the wider language network has implications for understanding both pre- and post-surgical naming in TLE.

## Highlights

• The health of fusiform-associated white matter affects naming ability in temporal lobe epilepsy.
• Healthier right hemisphere fusiform-associated white matter may be protective post-surgically.
• Some evidence for neurobiological distinction in auditory and visual naming outcomes.

## 1 Introduction

Object naming difficulties are among the most frequent cognitive complaints of patients with temporal lobe epilepsy (TLE) (Corcoran and Thompson, 1993) and present difficulty in day-to-day function. Further, naming abilities are at great risk following anterior temporal lobectomy (ATL), with significant declines reported in 25–60% of patients2, (Ives-Deliperi and Butler, 2012, Sherman et al., 2011). Given that surgery is a highly efficacious procedure for eliminating seizures (Jobst and Cascino, 2015), is cost-effective (Sheikh et al., 2020), and may prevent further neurodegeneration (Galovic et al., 2020), a better understanding of the cognitive barriers to extending surgery is important to matching TLE patients with treatment.

Naming is understood to be a complex process that simultaneously tests the function of multiple, interactive sub-systems of the language system: perceptual analysis, lexical-semantic processing, phonological processing, and speech articulation (Hamberger and Tamny, 1999, Indefrey and Levelt, 2004, Schuhmann et al., 2012). Current neurobiological models of language have linked these sub-systems to a distributed network of regions across the temporal, frontal, and parietal lobes (Poeppel et al., 2012, Price, 2012). Studies of naming impairment, induced either pre-surgically by epilepsy or post-surgically by resection, tend to focus on the importance of anterior and lateral temporal regions (Hamberger, 2015). A rich functional imaging literature has also identified numerous areas involved in naming, as well as broad temporal lobe regions that may contribute to naming impairment including posterior and ventral temporal regions (Fonseca et al., 2009, Trimmel, 2021, Farias et al., 2005, Trimmel, 2019).

Here we focused specifically on the role of basal-temporal language area (BTLA) (Lüders et al., 1991). Converging functional imaging, electrophysiology, and stimulation evidence has established the left BTLA, centered on the fusiform gyrus, as an important hub in both visual and auditory naming (Forseth et al., 2018). In addition, a basal-temporal region centered on the fusiform gyrus has emerged as an area of interest in the post-surgical literature. A recent symptom lesion mapping study demonstrated that resection of a left fusiform region increased risk for visual naming decline, regardless of the surgical approach (Binder et al., 2020). Additionally, a functional imaging study found correlations between pre-operative BOLD response in the fusiform and post-surgical visual naming outcome (Trimmel, 2019)**.** These studies highlight the importance of basal-temporal *cortex*, and possibly the fusiform specifically, to naming ability in TLE.

However, the BTLA is one of many nodes interconnected by a distributed white matter network subserving language ([Bibr b0100][Bibr b0105]). The integrity of this white matter is often disrupted due to ongoing seizures, which may lead to impairments in language ability, or diminish the possibility for successful post-surgical outcomes via limiting brain reserve capacity (Leyden et al., 2015, Balter et al., 2019). Therefore, we investigated whether the microstructural integrity of the white matter connecting the BTLA with the wider language network is associated with naming ability pre-surgically and/or predictive of post-surgical naming decline in TLE. Importantly, we test whether damage to this network disrupts both visual and auditory naming in a modality-independent manner, or whether it subserves visual naming alone, as differences have been identified in the activation patterns elicited by auditory versus visual naming (Trimmel, 2021, Farias et al., 2005)**.**

## 2 Methods

### 2.1 Standard protocol Approvals, Registrations, and patient consents

This study was approved by the Institutional Review Boards at the University of California, San Diego (UCSD) and University of California, San Francisco (UCSF) under a joint IRB plan. All participants provided informed consent according to the Declaration of Helsinki.

### 2.2 Participants

Fifty healthy controls and 88 patients with medically-refractory TLE (47 left-TLE; L-TLE and 41 right-TLE; R-TLE) had pre-surgical structural (sMRI) and diffusion-weighted (dMRI) imaging and neuropsychological measures of naming. Table 1 displays the demographics and clinical characteristics of the pre-surgical sample. Of these, 37 patients (19 L-TLE; 18 R-TLE) underwent ATL and had post-surgical naming data (see Supplementary Table 1). A TLE diagnosis was established by a board-certified neurologist with expertise in epileptology, in accordance with the criteria defined by the International League Against Epilepsy and based on video-EEG telemetry, seizure semiology, and neuroimaging evaluation. Presence or absence of hippocampal sclerosis was determined by visual inspection of MRIs by a board-certified neuroradiologist for detection of mesial temporal sclerosis (MTS). Patients with and without MTS were included, but patients were excluded if there was evidence of large structural lesions or visible extra-hippocampal pathology on clinical MRI. All patients completed pre-surgical imaging approximately a year prior to their surgery.

**Table 1:** Clinical and demographic characteristics for healthy controls (HC), left TLE (L-TLE), and right TLE (R-TLE) patients in the pre-surgical cohort.

	HC	L- TLE	R-TLE	test statistic
N	50	47	41	–
Age (years)	37.7 (14)	35.7 (13)	36.1 (13)	F(2;135) = 0.30; p = 0.74
Education (years)	15.8 (2.2)	13.5 (2)	13.6 (2.4)	F(2;135) = 16.9; p < .001
WTAR	117 (8.6)	97.7 (14)	93.1 (15)	F(2;135) = 41.7; p < .001
Sex (F/M)	20/30	22/25	17/24	FET; p = 0.78
Handedness (R/L)	46/3	40/7	39/1	FET; p = 0.097
Dominance (T/A)	47/3	35/12	39/2	FET; p = 0.0059
Age of Onset	–	20.9 (14)	21.2 (14)	t(86) = -0.103; p = 0.92
# current ASMs	–	2.21 (0.91)	2.35 (0.92)	t(85) = -0.698; p = 0.49
MTS (Y/N)	–	21/26	16/25	FET = 0.794; p = 0.67
Seizure Frequency#	–	8.36 (15)	10.2 (21)	t(85) = -0.459; p = 0.65

TLE: temporal lobe epilepsy; F: females; M: males; L: left; R: right; T: typical; A: a-typical; Y: yes; N: no; WTAR: Weschler’s Test of Adult Reading

Standard deviations are presented inside the parentheses.

# Seizure frequency includes both focal seizures as well as tonic clonic seizures.

### 2.3 Measures of naming

Visual object naming was evaluated with the Boston Naming Test (BNT (Kaplan et al., 2001)) and auditory naming was evaluated with the Auditory Naming Test (ANT (Hamberger and Seidel, 2003)). All participants had pre- and post-surgical naming scores on at least one of these two measures. They were tested prior to and an average of 15 months following ATL (mean: 15 months; std: 10 months). Of the 133 pre-operative patients and controls, 95% had BNT, 93% had ANT, and 88% had both test data. Of the 37 patients in the post-operative cohort, 92% had BNT, 92% had ANT, and 84% had both test data. Pre-to-post surgical naming change was calculated using reliable change indices that account for both measurement error (e.g., reliability of test) and for practice effects (RCI-PEs) (Maassen et al., 2009, Duff, 2012, Chelune et al., 1993, Iverson, 2001). Raw change scores were converted into RCI-PE z-scores to allow for direct comparison and a consistent interpretation between the two naming tests. For both naming measures, we used an updated method of calculating the standard error of the difference between test and re-test provided by Iverson et al. (Iverson, 2001) This enables a more accurate estimate as this formula additionally takes into account the variability in retest scores. For BNT, we used normative data derived from Sawrie et al. (Sawrie et al., 1996). For the ANT, we used normative data from Hamberger et al. 2003 (Hamberger and Seidel, 2003). RCI-PEs are reported as z-scores and used as continuous variables in all analyses.

### 2.4 Image acquisition

Imaging was performed on a General Electric Discovery MR750 3 T scanner with an 8-channel phased-array head coil at UCSD or UCSF. Image acquisitions were prospectively harmonized by ensuring acquisition was identical at both centers with identical protocols, scanner models, imaging sequences, and software versions. Acquisitions included a conventional three-plane localizer, GE calibration scan, a T1- weighted 3D structural scan (TR = 8.08 msec, TE = 3.16 msec, TI = 600 msec, flip angle = 8°, FOV = 256 mm, matrix = 256 × 192, slice thickness = 1 mm isotropic), and a single-shot pulsed-field gradient spin-echo EPI sequence (TE/TR = 96 ms/17 s; FOV = 24 cm, matrix = 128x128x48; axial). Diffusion-weighted images were acquired with b = 0 and b = 1000 mm^2^/s with 30 diffusion gradient directions. Two additional b = 0 volumes were acquired with either forward or reverse phase-encode polarity for use in B_0_ correction.

### 2.5 Image processing

*sMRI.* Automatic segmentation of the right and left hippocampus was performed with Freesurfer (v5.3) using the structural T1-weighted images. The segmentations were visually inspected to ensure correct labeling of the hippocampus. In order to control for differences in brain size, hippocampal volume (HCV) was divided by total intracranial volume for each participant.

*dMRI.* A detailed description of the preprocessing of the dMRI data is provided elsewhere (McDonald et al., 2014). In brief. DMRI images were corrected for spatial and intensity distortions due to B0 magnetic field inhomogeneities, eddy current distortion, gradient nonlinearity distortion, and head motion. The reverse gradient method was used to correct B0 distortion (Holland et al., 2010). A method using least squares inverse and iterative conjugate gradient descent was used to correct for eddy currents (Zhuang, 2006). Distortions due to gradient nonlinearity were corrected for each frame of the diffusion data (Jovicich et al., 2006). Head motion was corrected by registering each frame to the parameters obtained through diffusion tensor fitting, accounting for variation in image contrast across diffusion orientations (Hagler, 2009).

dMRI-derived fractional anisotropy (FA) was calculated based on a tensor fit to the b = 1,000 data.

*Fiber tract calculations.* Fiber tract FA values were derived using a probabilistic diffusion tensor atlas (i.e. AtlasTrack) that has been validated in healthy controls and patients with TLE (Hagler, 2009). AtlasTrack is a fully automated method for labeling fiber tracts in individual subjects based on diffusion-weighted images, T1-weighted images, and a probabilistic atlas of fiber tract locations and orientations. For each participant, the T1-weighted structural images were nonlinearly registered to a common space and the respective diffusion tensor orientation estimates were compared to the atlas. This resulted in a map of the relative probability that a voxel belongs to a particular tract given its location and similarity of diffusion orientation. Voxels identified with Freesurfer 5.3.0 as CSF or gray matter were excluded from the fiber regions of interest (ROIs). Average FA was calculated for each fiber ROI, weighted by fiber probability, so that voxels with low probability of belonging to a given fiber contributed minimally to average values. A full description of the atlas and detailed steps used to create the atlas are provided elsewhere (Hagler, 2009).

*Superficial White Matter (SWM) calculations.* Cortical surface reconstruction and parcellation was determined using FreeSurfer and the Desikan-Killiany atlas. FA for SWM was calculated by sampling the white matter below the pial surface at each vertex (Salat et al., 2009). Surface cortical parcellations were subsequently used to assign a label to the underlying WM by the construction of a Voronoi diagram in the WM voxels based on distance to the nearest cortical parcellation label (Salat et al., 2009)**.** Using this label, average FA was measured within the white matter beneath the fusiform ROIs in each individual’s native space. For the surface-based analyses, group maps were created by resampling individual surfaces into a common spherical coordinate system that aligned cortical folding patterns across participants and each individuals’ data was smoothed with a 16 mm Gaussian kernel (Fischl et al., 1999)**.** ROI averages were computed on unsmoothed data to ensure that average values are localized within the ROI and not influenced by neighboring regions in accordance with previous studies examining SWM (Reyes et al., 2019).

### 2.6 Selection of tracts and ROIs

**Fig. 1:**
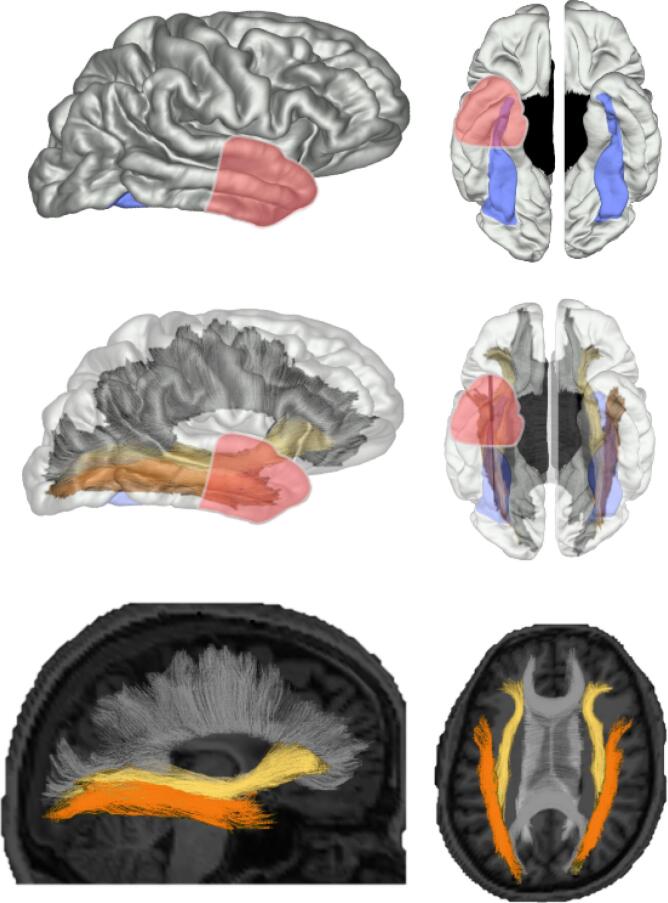
**Illustration of approximate anterior temporal lobectomy (ATL) extent, deep white matter (WM) tracts, and superficial WM (SWM) fusiform ROI.**Top row) Sagittal and ventral view of the SWM ROI of the fusiform region (violet) derived from the Desikan-Killiany Atlas and an illustration of the approximate extent of an ATL performed on the left hemisphere. Middle row) Overlay of the approximate ATL extent illustration displayed over the inferior longitudinal fasciculus (ILF; orange) and inferior frontal occipital fasciculus (IFOF; yellow). Bottom row) Sagittal and coronal views of the ILF and IFOF derived from AtlasTrack and projected onto a T1-weighted image for a single individual. The corpus callosum is portrayed in light gray in order to provide additional spatial information. (For interpretation of the references to color in this figure legend, the reader is referred to the web version of this article.)

The inferior longitudinal fasciculus (ILF) and inferior frontal occipital fasciculus (IFOF) were selected due to evidence that they are often affected in TLE (McDonald et al., 2008), contribute to pre-surgical naming performance in TLE (Leyden et al., 2015), and course through the inferior temporal lobe with connections to basal temporal cortex and the fusiform specifically (Palejwala, 2020). The ILF is a long-range association tract that connects the fusiform and lingual areas in the temporo-occipital junction to anterior temporal cortex (Panesar et al., 2018). The IFOF is a long-range association tract that connects the fusiform and lingual areas with frontal cortex including the inferior frontal gyri (Wu et al., 2016). The top row of Fig. 1 displays the SWM fusiform ROI that was selected based on its association with pre-surgical naming (Forseth et al., 2018) as well as post-surgical naming outcomes (Binder et al., 2020). We also selected additional volumes (amygdala), tracts (cortico-spinal tract, which connects motor cortex and the spinal cord), and SWM regions (entorhinal) which were not associated with language in prior studies to ensure our findings were not related to generalized FA effects throughout the brain (see Supplementary Fig. 1). These additional volumes, tracts, and SWM regions were defined using the same methods as detailed above.

### 2.7 Statistical analysis

One-way ANOVAs, independent samples t-tests, and Fisher’s exact tests were conducted to examine differences in demographic and clinical variables among HC, L-TLE, and R-TLE patients (Table 1 and Supplementary Table 1). Fisher’s exact and independent samples *t*-tests tested differences in naming RCI-PEs between groups. Spearman’s bivariate correlations examined associations between RCI-PEs and clinical, demographic, and imaging variables. We note when *p*-values survived a 5% FDR correction (Benjamini and Hochberg, 1995). Because our main focus is on imaging variables of neurobiology and clinical variables were included for comparison with previous studies, we FDR corrected separately for clinical (N = 8) and imaging (N = 5) variables. Given the larger number of tracts and regions, the FDR correction was more conservative for the imaging variable set. We examined correlations separately for L-TLE and R-TLE to assess the differential impact of left versus right ATL on naming performance.

Because a-typical (i.e., bilateral or right hemisphere) language dominance has been shown to influence risk for naming decline (Binder, 2011), we also re-ran all correlations including only left language dominant patients. Language dominance was based on Wada results and/or fMRI language laterality when available. When neither Wada nor fMRI was available, handedness was used (i.e., left language dominance was inferred from right handedness) as has been the approach in prior studies (Busch et al., 2018). For the pre-surgical cohort, Wada was available for 15%, fMRI available for 57%, and handedness was used for 28%. For the post-surgical cohort, Wada was available for 38%, fMRI available for 41%, and handedness was used for 21%.

**Fig. 2:**
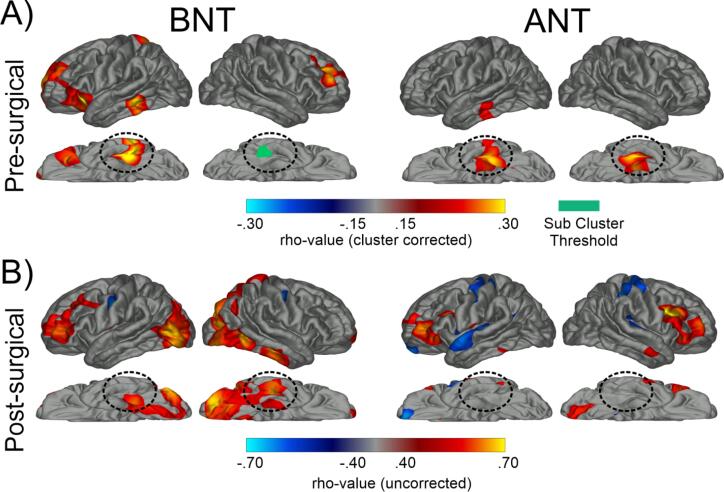
**Whole-brain superficial white matter correlations with naming. A)** Whole-brain, cluster-corrected superficial white matter correlations with pre-surgical Boston Naming Test (BNT) and Auditory Naming Test (ANT) performance. Blue denotes negative correlations while red/yellow denotes positive correlations. All maps are cluster corrected at *p* < .05. The dashed, circled area highlights basal-temporal superficial white matter. Teal denotes a cluster of reduced FA in the basal-temporal superifical white matter that was sub-threshold after cluster correction. **B)** Whole-brain, uncorrected superficial white matter correlations with post-surgical Boston Naming Test (BNT) and Auditory Naming Test (ANT) RCI-PEs**.** Blue denotes negative correlations while red denotes positive correlations. Circled areas represent basal-temporal superficial white matter. (For interpretation of the references to color in this figure legend, the reader is referred to the web version of this article.)

For the surface-based analyses, relationships with naming variables were determined using vertex-wise Spearman correlations. Statistical correction was applied using cluster-based thresholding (Worsley et al., 1999) (cluster-corrected *p* < .05). Due to the small post-operative LTLE sample size, Fig. 2B was not p-value cluster corrected and should be regarded as preliminary and hypothesis generating.

## 3 Results

### 3.1 Patient demographics and clinical variables

In the pre-surgical sample, groups did not significantly differ on demographic or clinical variables, except for education, estimated intellectual function (i.e., WTAR reading score), and language dominance (Table 1). HC had significantly more education and higher WTAR score than L- & R-TLE (both *p* < .001) and L-TLE had more a-typical language dominant patients than the other groups (*p* < .01). In the post-surgical sample, L- and R-TLE did not differ in age, education, sex, handedness, age of onset, number of current anti-seizure medications (ASMs), MTS status, seizure frequency (combined focal seizures and generalized tonic-clonic seizures), Engel outcome, or language dominance (all *ps* > 0.05; see Supplementary Table 1 for means and statistical tests).

### 3.2 Pre- and Post-surgical naming performance

Table 2 shows average pre- and post-surgical naming scores, as well as the change scores (i.e., RCI-PEs), and percentage of patients who showed clinically significant decline on the BNT and ANT for L-TLE and R-TLE patients. Supplementary Figure 2 plots individual data points. For the pre-surgical group, a one-way ANOVA revealed significant group differences for both ANT and BNT (*ps* < 0.001). Follow-up tests demonstrated that HC had significantly better naming performances than L-TLE (BNT: *p* < .001, ANT: *p* < .001) and R-TLE (BNT: *p* < .001, ANT: *p* < .001), whereas L- and R-TLE did not differ (BNT: *p* = .12, ANT: *p* = .40). Post-surgically, the L-TLE showed a group-level decline on ANT [t(17) = -2.6; *p* = .02] but not BNT [t(15) = -0.90; *p* = .38]; R-TLE did not show group-level decline in BNT [t(17) = 0.39; *p* = .7] or ANT [t(15) = 0.06; *p* = .95]. The magnitude of the decline did not differ between L-TLE and R-TLE for BNT [t(32) = -0.98; *p* = .34] or ANT [t(32) = -1.76; *p* = .09]. At an individual level, the number of decliners was numerically greater in L-TLE but did not significantly differ between L- and R-TLE for BNT (38% vs 16%, respectively; FET = 2.9; *p* = .25) or for ANT (33% vs 13%, respectively; FET = 3.38; *p* = .23).

**Table 2:** Pre-surgical and post-surgical scores, as well as change scores and percent of patient who showed significant decline on neuropsychological tests of naming post-surgically.

	HC	Left TLE	Right TLE	Group Comparison	
Boston Naming Test					
Pre-surgical raw scores(Total Sample)	56.1 (2.8)	45.2 (9.3)	47.9 (9.2)	F(2;129) = 26.5; p < .001	
Post-surgical raw scores(Post-surgical sample)	–	45.3 (7.6)	49.8 (8.7)	–	
Change (RCI-PE)	–	−0.48 (2.1)	0.15 (1.6)	t(32) = -0.975; p = 0.34	
Auditory Naming Test					
Pre-surgical(Total Sample)	49.3 (0.91)	46.3 (4)	46.8 (4.9)	F(2;125) = 8.38; p < .001	
Post-surgical(Post-surgical sample)	–	45.7 (3.6)	48.3 (2.0)	–	
Change (RCI-PE)	–	−1.13 (1.8)	0.03 (2.0)	t(32) = -1.76; p = 0.088	

TLE: temporal lobe epilepsy; RCI-PE: reliable change indices that account for practice effects; Y: yes; N: no

### 3.3 Relationship with Pre-Surgical naming ability

Fig. 2A displays whole brain, vertex-wise maps of the correlations between SWM FA and pre-surgical BNT and ANT scores. Higher scores on both tests were associated with higher FA in the left basal-temporal SWM, centered on the fusiform. Within the right hemisphere, ANT showed a significant cluster in the fusiform, whereas BNT had a cluster which though present, failed to survive multiple comparison correction. Higher BNT scores were also associated with higher FA within lateral prefrontal SWM bilaterally, including part of the inferior frontal gyrus, anterior insula, and the middle frontal gyrus, and both higher BNT and ANT naming scores showed an association with a small region of SWM within the lateral left temporal lobe.

Table 3 shows correlations between naming ability and demographic, clinical, and imaging variables.

**Table 3:** Spearman bivariate correlations between pre-surgical naming scores and clinical as well as imaging variables for the combined group (controls, L-TLE, & R-TLE).

	BNT	ANT
Education	0.43**	0.39**
Age	0.13	0.22*
Age of Seizure Onset	0.36**	0.24*
Duration	−0.19	0.02
ASMs #	0.14	−0.007
L-Hippocampus (Volume)	0.19*	0.16
R-Hippocampus (Volume)	−0.037	0.026
L-ILF (FA)	0.24**	0.33**
R-ILF (FA)	0.23**	0.33**
L-IFOF (FA)	0.31**	0.27**
R-IFOF (FA)	0.37**	0.36**
L-Fusiform (FA)	0.3**	0.33**
R-Fusiform (FA)	0.16	0.14

Significant effects that survived FDR correction are bolded.

TLE: temporal lobe epilepsy; BNT: boston naming test; ANT: auditory naming test; ASMs: anti-seizure medications; ILF: inferior longitudinal fasciculus; IFOF: inferior longitudinal fasciculus; L: left; R: right

* p < .05;

** p < .01;

*Clinical and demographic variables correlated with pre-surgical naming ability.* Higher education [BNT: r(135) = 0.43; *p* < .001; ANT: r(132) = 0.39; *p* < .001] and older age of seizure onset [BNT: r(86) = 0.36; *p* < .001; ANT: r(89) = 0.24; *p* = .022] correlated with higher pre-surgical BNT and ANT scores. In addition, older age was associated with higher ANT scores [r(132) = 0.22; *p* = .012]. All significant correlations survived FDR correction.

**Fig. 3:**
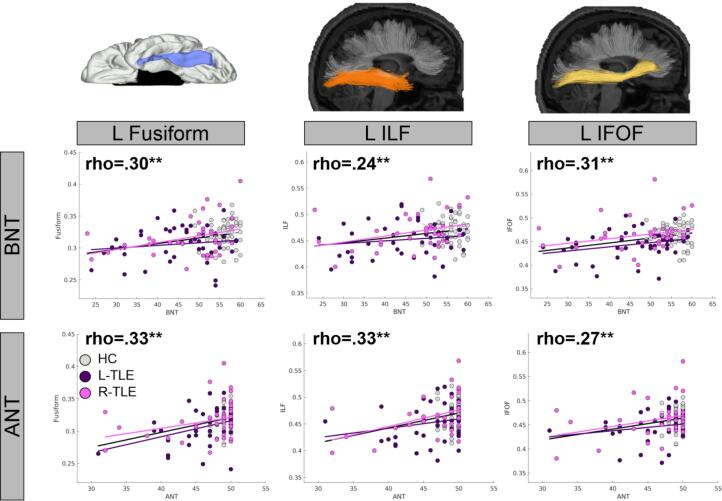
**Associations between basal-temporal FA values and pre-surgical naming ability.** Scatterplots depict the relationship between FA of the left fusiform (L Fusiform), left inferior longitudinal fasciculus (L ILF), and left inferior frontal occipital fasciculus (L IFOF) and Boston Naming Test (BNT) and Auditory Naming Test (ANT). Dots denote individual patients from the Left TLE (L-TLE; purple), Right TLE (R-TLE; pink), and healthy controls (HC; grey). Black line denotes overall trend line associated with rho-value, with additional lines for each individual groups color added to show consistency of relationship across groups. Significant effects that survived FDR correction are bolded (all displayed values survived FDR). * p < .05; ** p < .01. (For interpretation of the references to color in this figure legend, the reader is referred to the web version of this article.)

*Imaging variables and pre-surgical naming ability.* A greater L-HCV was associated with higher BNT scores [r(135) = 0.19; *p* = .024], whereas the association with ANT scores approached significance [r(132) = 0.16; *p* = .062]. Bilaterally, both tracts were associated with higher naming ability: L-ILF [BNT: r(130) = 0.24; *p* = .007; ANT: r(127) = 0.33; *p* < .001], R-ILF [BNT: r(130) = 0.23; *p* = .008; ANT: r(127) = 0.33; *p* < .001], L-IFOF [BNT: r(130) = 0.31; *p* < .001; ANT: r(127) = 0.27; *p* = .002], and R-IFOF [BNT: r(130) = 0.37; *p* < .001; ANT: r(127) = 0.36; *p* < .001]. For the SWM, higher L-Fusiform FA was associated with higher BNT [r(130) = 0.30; *p* < .001] and higher ANT [r(127) = 0.33; *p* < .001] scores. Neither the R-HCV nor the R-Fusiform were associated with higher naming scores (all *p*s > 0.05). Fig. 3 displays scatterplots illustrating the correlations. Supplementary Table 2 displays correlations for additional ROIs and tracts not typically associated with naming, demonstrating that correlations with higher naming scores were not universal, and had some specificity to our a-priori selections. All significant correlations survived FDR correction.

*Typical Language Dominance only.* Including only left language-dominant patients did not meaningfully change the pattern of these results (see Supplementary Table 3). The only notable changes were that the correlation between left HCV and BNT and between the left IFOF FA and ANT no longer reached FDR-corrected significance.

### 3.4 Relationship with Post-surgical naming change

Table 4 shows correlations between RCI-PEs and demographic, clinical, and imaging variables, separately for L-TLE and R-TLE.

**Table 4:** Spearman bivariate correlations between pre- to post-surgical naming change scores and clinical as well as imaging variables for a) post-surgical L-TLE & b) post-surgical R-TLE.

	L-TLE			R-TLE	
	BNT	ANT		BNT	ANT
Pre-surgical Score	−0.5*	−0.26		−0.063	−0.7**
Education	0.22	0.19		−0.45	−0.13
Age	−0.19	−0.16		0.034	−0.64**
Age of Seizure Onset	−0.26	−0.28		−0.21	−0.23
Duration	0.16	0.15		0.14	−0.29
# ASMs	−0.37	−0.25		0.13	0.21
L-Hippocampus (Volume)	−0.55*	−0.34		−0.13	−0.16
R-Hippocampus (Volume)	0.21	0.28		−0.16	−0.23
L-ILF (FA)	0.55*	0.45		0.54*	0.044
R- ILF (FA)	0.33	0.042		0.32	−0.28
L- IFOF (FA)	0.34	0.54*		0.3	−0.48
R- IFOF (FA)	0.52*	0.11		0.51*	−0.2
L-Fusiform (FA)	0.21	−0.16		0.28	−0.3
R-Fusiform (FA)	0.72**	0.28		−0.03	−0.32

Significant effects that survived FDR correction are bolded.

TLE: temporal lobe epilepsy; BNT: boston naming test; ANT: auditory naming test; ASMs: anti-seizure medications; ILF: inferior longitudinal fasciculus; IFOF: inferior longitudinal fasciculus; L: left; R: right

* p < .05;

** p < .01;

*Clinical and demographic variables and post-surgical naming change. LTLE.* A higher pre-surgical naming score was associated with greater decline on BNT [r(14) = 0.5; *p* = .048] but not ANT [r(16) = 0.26; p = .3]. However, this did not survive FDR correction. *RTLE.* A higher pre-surgical naming score was associated with greater ANT decline, [r(14) = 0.7; *p* = .0024] as was a higher age at surgery [r(14) = 0.64; *p* = .007], both of which survived FDR correction.

**Fig. 4:**
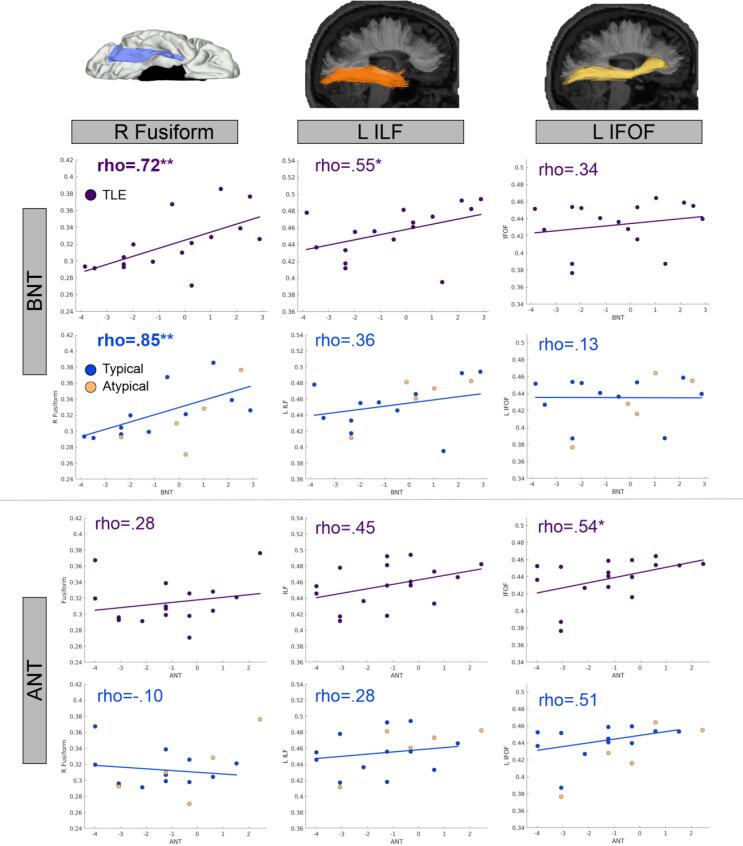
**Associations between basal temporal FA values and post-surgical naming change in patients with left TLE (L-TLE).** Scatterplots depict the relationship between FA of the right fusiform (R Fusiform), left inferior longitudinal fasciculus (L ILF), and left inferior frontal occipital fasiculus (L IFOF) and Boston Naming Test (BNT) and Auditory Naming Test (ANT) in L-TLE. Top row for each test shows the relationship between variables for all L-TLE’s (purple). The bottom row for each test shows the relationship for L-TLE split into typical (blue) and a-typical (light orange) patients. Colored lines and rho-values denotes trend line associated with the groups. Significant effects that survived FDR correction are bolded. * p < .05; ** p < .01. (For interpretation of the references to color in this figure legend, the reader is referred to the web version of this article.)

*Imaging variables and post-surgical naming change. LTLE.* Higher left HCV was associated with greater post-surgical decline for BNT [r(14) = 0.55; *p* = .028] but not for ANT [r(16) = 0.34; *p* = .17]. For the white matter tracts, the opposite pattern was observed, with higher FA generally associated with *less* decline, but only some correlations reached significance. For BNT, higher L-ILF [r(14) = 0.55; *p* = .028] and R-IFOF [r(14) = 0.52; *p* = .037] FA was associated with less decline. For ANT, higher FA of the L-IFOF [r(16) = 0.54; *p* = .027] was associated with less decline. For the SWM, higher FA of the R-Fusiform was associated with less decline on the BNT [r(14) = 0.72; *p* = .0018]. Scatterplots depicting these relationships are shown in Fig. 4 (top rows). In addition, preliminary whole brain correlation maps (uncorrected) are presented in Fig. 2B. For LTLE, only the relationship between BNT and the R-Fusiform survived FDR correction. *RTLE.* Higher FA of the L-ILF [r(14) = 0.54; *p* = .031] and R-IFOF [r(14) = 0.51; *p* = .042] was associated with less BNT decline, but these did not survive FDR correction.

*Typical language dominance only.* To determine whether language dominance was driving the positive associations between white matter and post-surgical scores, patients were divided into those with typical and a-typical language dominance. As can be seen in Fig. 4 (bottom rows), after removing the a-typical (right-dominant or bilateral) language dominant patients and including only patients with typical language dominance, none of the long-range fiber associations reached significance in LTLE patients, although the direction of the relationships remained the same even if the strength of the relationship was attenuated. The only correlation that survived FDR-correction with this reduced sample size was the association between higher FA of the R-Fusiform and less post-surgical decline on the BNT [r(9) = 0.84, *p* < 0.001].

## 4 Discussion

Naming difficulties are often attributed to anterior and/or posterior-lateral temporal dysfunction in TLE (Hamberger, 2015), caused pre-surgically by epilepsy-driven factors or post-surgically by resection/ablation. Here, we demonstrate that healthier microstructural integrity of white matter in the basal-temporal lobe is associated with better naming ability pre-surgically and a higher chance for successful post-surgical naming outcome. Pre-surgically, higher microstructural integrity in the left fusiform and bilateral white matter tracts that connect the fusiform to anterior temporal (i.e., ILF) and prefrontal cortex (i.e., IFOF), were associated with higher naming performance. Importantly, this pattern was observed for both visual and auditory naming, suggesting a-modal contributions to naming. Post-surgically, greater microstructural integrity of the white matter within the contralateral (right) fusiform correlated with better visual naming outcomes in L-TLE, suggesting strong structural reserve capacity of this region. These interesting preliminary findings suggest that integrity of the basal-temporal white matter within the ipsilateral and contralateral hemispheres may contribute to naming in TLE, though future studies will be needed to confirm and extend our post-operative findings.

### 4.1 Basal-temporal white matter supports naming ability

Successful naming involves many distributed subcomponent processes (Hamberger and Tamny, 1999, Indefrey and Levelt, 2004, Schuhmann et al., 2012), requiring a broad, interconnected white matter network (Kaestner, 2020, Kljajevic, 2014). Naming impairment could result from localized white matter damage disrupting any of the specific modules of the language system (Hamberger et al., 2010), or from widespread disconnection between modules driven by damage to long range association tracts (McDonald et al., 2008). Our investigation found both localized and distributed relationships with naming ability in the basal-temporal lobe. Locally, impaired naming was associated with microstructural damage to the white matter abutting the left fusiform (i.e., SWM). This SWM beneath the cortex is comprised of U-fibers interconnecting neighboring neuronal populations and has been shown to be particularly important for cognition given its role in maintaining cortical–cortical connectivity (Nazeri, 2015, Ouyang et al., 2017). Furthermore, SWM is sensitive to TLE-related pathology and may be an important predictor of post-surgical seizure outcomes (Liu et al., 2016). At the whole-brain level, a fusiform-centered SWM region was also identified that was broadly homologous to the a-modal naming region identified by converging fMRI, iEEG, and stimulation approaches (Forseth et al., 2018). This region of the mid-fusiform has been associated with both visual and auditory language (Forseth et al., 2018, Spitsyna et al., 2006, Marinkovic et al., 2003), acting as one of the heteromodal hubs that links symbols such as words, images, and sounds with meaning. Given that impaired lexical-semantic processing may contribute to naming impairments in TLE ((Bell et al., 2001)Giovagnoli et al., 2005), disruption in the basal-temporal white matter may be a key contributor to this impairment. The exploratory whole-brain SWM analysis for post-operative outcomes in BNT (see Fig. 2B) also implicated other expected language regions such as the inferior and middle frontal gyri in addition to the basal temporal region.

In addition, two long-range association tracts that interconnect the basal-temporal regions with the anterior temporal (ILF) and dorsolateral prefrontal (IFOF) region were associated with pre-surgical naming ability. These two tracts are theorized to be complementary, and together comprise a multi-component ventral visual route interconnecting a network of regions supporting lexical-semantic processing (Duffau et al., 2013). The IFOF, is a ‘direct’ route, playing a role in semantic processing via direct connections of basal-temporal areas with frontal and temporal-parietal cortex, with stimulation leading to disrupted semantic processing (e.g., semantic paraphasias) (Duffau, 2005). The ILF is an ‘indirect’ route, connecting basal-temporal areas with the anterior temporal lobe which is relayed to frontal and lateral temporal-parietal regions through additional white matter connections (Herbet et al., 2018). Disruption of the ILF has been associated with lexical-level word finding difficulties (Herbet et al., 2016). Here, the integrity of both tracts was found to have strong, positive relationships with auditory and visual naming ability pre-surgically, suggesting that they may share a role in amodal processing of lexical-semantic information (Duffau et al., 2013).

### 4.2 Basal-temporal white matter supports successful post-surgical outcomes, especially in visual naming

Successful naming post-surgically depends on both functional adequacy and functional reserve. Functional adequacy reflects whether the tissue being resected or transected supports critical language function, with individuals with healthier tissue and higher ability pre-surgically at greater risk for post-surgical decline (Busch et al., 2018, Chelune, 1995). On the other hand, functional reserve refers to the capacity of the residual (non-resected) tissue, to support language function following surgery or injury to the dominant hemisphere. Here, L-TLE patients with a healthier hippocampus as well as a higher pre-surgical naming score (high functional adequacy) were at high risk for decline in visual naming post-ATL.

Conversely, for the white matter, a more complicated pattern emerged. For long-range fibers, when the entire L-TLE group was analyzed together, in addition to higher FA values in the to-be-spared right IFOF being associated with less decline, higher FA values in the left hemisphere for the ILF and IFOF were associated with less decline, seemingly contrary to the functional adequacy hypothesis. However, splitting the group by language dominance revealed that once patients with a-typical language were removed, this unexpected left-hemisphere pattern was somewhat attenuated. When only patients with left (typical) language dominance were included, while the long-range fibers relationships remained positive, these relationships with naming did not reach significance. This is likely due to a combination of factors, including both a decreased sample size and a weaker relationship, both due to removal of 5 patients with a-typical language. Although it is not possible to fully disentangle the contribution of these two factors with our sample size, visual inspection of our post-operative scatterplots (Fig. 4) suggests that patients with a-typical language did augment the left-hemisphere correlations. This could be because long-range fibers may be particularly important to successful post-surgical outcomes in the presence of a-typical language dominance due to mediating a potentially more widely distributed and heterogeneous language network. In a-typical language re-organization, it is often not clear whether increased rightward hemisphere involvement reflects a homologous right-lateralized language network (Knecht, 2003) or a more distributed combination of left and right regions (Dijkstra and Ferrier, 2013). In TLE, a fMRI study of 80 a-typical dominance patients found only 17 had a clearly right-lateralized pattern; the remaining 63 had a mix of bilaterality, left, and right activations (Berl, 2014). Future study with a larger sample size will be necessary to fully appreciate the importance of long-range fiber tracts to post-surgical naming outcomes in patients with typical language organization.

The only consistent pattern across both the typical language and the pooled L-TLE patient group was the association between greater integrity in the *right* hemisphere fusiform SWM and better visual naming outcomes. Although our findings suggest the importance of the right fusiform post-surgically (i.e. the potential functional reserve of this region), the mechanisms through which this develops are unclear. Pre-surgically, some amount of re-organization to the right hemisphere may occur in patients with left TLE secondary to left hemisphere pathology or developmental factors (Dijkstra and Ferrier, 2013, Stewart et al., 2014). Functional imaging studies have identified that a rightward shift is protective for post-operative naming in LTLE, whereas strong left-sided language activations is a risk factor for decline (Trimmel, 2019, Binder, 2011, Rosazza, 2013). For example, one study examining hemispheric language dominance using an ROI including lateral, medial, and ventral temporal regions found that stronger leftward activation was correlated with greater decline in both visual and auditory naming (Trimmel, 2019). The relationship between right-lateralized function and a concomitant right-lateralized structure is supported by data showing that right-shifted language re-organization in L-TLE pre-surgically (measured with fMRI) is associated with a rightward shift in perisylvian white matter integrity and better pre-surgical language performance (Chang, 2017). Another possibility is that, post-surgically, better visual naming scores rely on the functional reserve of the contralateral fusiform (Chelune, 1995) (i.e., greater integrity of the right fusiform), enabling patients to better utilize this region as the language network adapts. Longitudinal studies with larger samples will be necessary to determine the degree to which functional reorganization, functional reserve and/or another mechanism drives the importance of the right basal-temporal region to post-surgical naming outcomes.

### 4.3 The relationship between auditory and visual naming

The large majority of studies of pre-surgical (Hamberger, 2015) and post-surgical (Sherman et al., 2011) naming focus on visual naming only. While some theories posit that a high percentage of the language network is a-modal (i.e., sustains the same cognitive operation of language processing regardless of input modality), research is still divided on the total proportion of the overlap (Kaestner et al., 2021). Therefore, it is not clear how generalizable visual naming findings are to auditory naming, and perhaps to auditorily-encoded language more generally. Here, pre-surgically we found strong overlap in the relationship between auditory and visual naming performance and basal-temporal white matter integrity. This is consistent with reports that basal-temporal regions (e.g., BTLA) is an important a-modal hub of lexical-semantic processing (Forseth et al., 2018), with our findings extending the overlap beyond the cortex into the surrounding white matter network.

A different pattern emerged post-surgically. The relationship of basal-temporal white matter to visual naming decline was stronger and more consistent than the relationship with auditory naming decline. It is not clear if this is due to our smaller post-surgical sample size, or psychometric properties of the tests (e.g., varied difficultly level across measures). A previous report of the importance of the basal-temporal area to surgical outcomes was based on visual naming only (Binder et al., 2020); and some functional imaging studies of post-operative naming have also focused on visual naming alone (Binder, 2011, Rosazza, 2013). Interestingly, a functional imaging study that did include both visual and auditory naming reported a dissociation—pre-operative BOLD response in the fusiform correlated with visual naming outcome, whereas pre-operative BOLD response in the inferior temporal gyrus correlated with auditory naming outcome (Trimmel, 2019). This would suggest that it is possible for a region to only to be *critical* for one modality post-surgically. This would reflect differences in the processing streams for auditory and visual language. There are multiple posited lexical-semantic hubs in the brain, including the temporal pole, posterior middle-temporal gyrus, and possibly the fusiform (Price, 2012). Given this distributed nature of lexical-semantic processing in the brain, the importance of any specific hub is dictated by its specific relationship to the input language modality. This is further illustrated by the differences in functional activation patterns elicited by auditory and visual naming (Trimmel, 2021, Farias et al., 2005). Given the importance of the ventral processing stream to visual orthographic language as well as visual objects (Thesen, 2012, Nobre et al., 1994), our hypothesis is that the current results relate to the privileged role of the ventral temporal regions in visual mediated language. Continued study will be necessary to determine the generalizability of the benefits of sparing this ventral region to language.

Our study did not delve into sparing vs resecting of BTLA. Rather, we investigated how pre-surgical white matter network integrity affected outcomes in the context of a standard ATL surgical approach. Our whole-brain post-operative SWM, which should be considered preliminary and hypothesis generating, hinted that for BNT, correlations were present bilaterally in the ventral temporal lobe, more anterior in the (non-resected) right hemisphere than the left. The ANT maps were weaker and no consistent patterns emerged in the temporal lobe; however, positive correlations were present in the bilateral inferior frontal regions (Fig. 2B). This matches well with studies of the naming network pre-surgically, which show that in fMRI activations and iEEG electrodes, visual naming is more prevalent in ventral temporal areas whereas auditory naming is more prevalent in prefrontal areas (Forseth et al., 2018). Given that the total overlap of auditory and visual naming is unknown, but that modality-specific differences in the overall network have been identified (Trimmel, 2021, Farias et al., 2005), it is possible that relationships between SWM and post-surgical outcomes may be modality-specific, even if they overlap pre-surgically.

Finally, the role of the hippocampus in naming is debated. One prominent theory suggests it is involved in visual but not auditory naming (Hamberger et al., 2010). Our observations were somewhat consistent with this position. Here we found that pre-surgically, left HCV was significantly associated with visual naming, whereas the correlation with auditory naming only approached significance. Post-surgically, removal of a visually intact left hippocampus was a significant risk factor in visual but not auditory naming decline for L-TLE. The role of the hippocampus in naming was questioned by earlier studies which found that hippocampal amnesia did not impair naming ability (Kensinger et al., 2001); however, more nuanced studies found that hippocampal damage reduces the depth of semantic knowledge associated with words (Klooster and Duff, 2015, Hilverman and Duff, 2021), and involvement of the hippocampus in naming has been found during iEEG recordings (Hamamé et al., 2014). Further study is necessary to elucidate the mediating role of the hippocampus in naming (Duff et al., 2020).

### 4.4 Limitations & future directions

The main limitation in this study is the sample size of our post-surgical sample, which limited our ability to consider all the possible factors that may contribute to post-surgical naming decline. Second, in this study we focused on an ATL-only population, which provided a consistent surgical approach, but does not answer questions regarding the possibility of tailoring surgery to improve naming outcomes. As surgical interventions evolve toward greater spatial precision and targeted treatment(Gross et al., 2015, Gross, 2018) it will be important to use spatially-precise techniques (e.g., lesion-mapping approaches) to examine how white matter sparing may preserve surgical outcomes. Thirdly, our superficial white matter ROI covered the full extent of the fusiform; future studies may subdivide this region to achieve a more full spatial characterization including a comparison of areas resected and preserved during an ATL. Fourthly, our language laterality was based on handedness in a subset of patients who did not have fMRI or Wada available. However, this approach has also been taken in other multi-center studies of post-surgical naming outcomes (Busch et al., 2018). Lastly, we did not have consistent stimulation mapping available to identify whether sparing vs removal of BTLA influenced naming outcomes.

A final observation is that the decline in visual naming in our LTLE cohort was more subtle than is reported in many prior studies (Trimmel, 2019, Binder et al., 2020, Busch et al., 2016) and the magnitude of decline in our LTLE and RTLE patients did not significantly differ (Busch et al., 2016, Schwarz and Pauli, 2009, Kovac et al., 2010). In our cohort, these observations were driven by the rather modest decline observed in our LTLEs at the group level, though a visual examination of Supplementary Figure 2 shows that our LTLE patients achieved a range of naming outcomes which included some strong declines. There are several possible explanations. First, studies which report substantial naming declines often test patients 4–7 months post-surgery (Binder et al., 2020, Busch et al., 2016, Trimmel, 2019) whereas our patients were tested an average of 15 months post-surgically. Given that greater naming declines may occur earlier in the postoperative period (Langfitt and Rausch, 1996) and that two studies report decline in the first 6 months followed by an improvement in naming scores at the one year mark (Abdallah, 2021, Giovagnoli, 2016), it is possible that our cohort includes patients who have successfully adapted to the effects of surgery through re-organization of language networks or other compensatory mechanisms. A second possibility is that our cohort differs from previously reported surgical cohorts. This could be due to a potentially more conservative surgical approach at our participating centers or a more modern patient sample (all surgeries in this study were performed after 2011) which could lead to better overall naming outcomes. Finally, we note that our ANT results are closer to the expected pattern, as the LTLE showed a significant group-level post-surgical decline that was also marginally lower than RTLE (*p* = .09). A longitudinal study of naming with testing at multiple post-operative time points will be necessary to fully understand how time since surgery and neurobiology interact to affect post-operative naming outcomes at an individual patient level.

Combining the post-surgical lesion mapping approach of Binder and colleages(Binder et al., 2020) with the multimodal pre-surgical mapping approach of Forseth et al. (Forseth et al., 2018) would allow for a comprehensive exploration of the BTLA and surrounding basal-temporal region, and clarify their relationship to naming outcomes. Further, understanding the exact role of the ventral temporal lobe in language could benefit from additional investigation. For example, here we focused on naming and white matter directly related to the fusiform. However, future studies could include dorsal white matter tracts, such as the arcuate fasciculus, which is well-known to be involved in language, as well as other language domains, including fluency or vocabulary to more fully characterize ventral temporal contributions to language. Our study took a more targeted approach to demonstrate the importance of basal-temporal white matter networks to successful naming, both pre- and post-surgically. These results could guide future, well-powered studies that expand upon and translate these findings into clinical practice.

## Declaration of Competing Interest

The authors declare that they have no known competing financial interests or personal relationships that could have appeared to influence the work reported in this paper.
