# Supplementary material for: The importance of basal-temporal white matter to pre- and post-surgical naming ability in temporal lobe epilepsy
Source: Neuroimage Clin. 2022 Feb 9;34:102963. doi: 10.1016/j.nicl.2022.102963 (PMC8888987; doi:10.1016/j.nicl.2022.102963)
Supplement: Supplementary data 1 [file mmc1.docx]

**Supplementary Table 1.** Clinical and demographic characteristics for left TLE (L-TLE) and right TLE (R-TLE) patients that underwent ATL.

|  | L- TLE | R-TLE | test statistic | |
| --- | --- | --- | --- | --- |
| N | 19 | 18 |  | |
| Age (years) | 34.6 (14) | 33.8 (12) | t(35)=0.178; p=0.86 | |
| Education (years) | 13.9 (2) | 13.9 (2.6) | t(35)=0.0766; p=0.94 | |
| **WTAR** | **97.7 (14)** | **93.1 (15)** | **t(35)=0.991; p=0.33** | |
| Sex (F/M) | 6/13 | 6/12 | FET=1.08; p=1 | |
| Handedness (R/L) | 15/4 | 18/0 | FET=Inf; p=0.11 | |
| Dominance (T/A) | 14/5 | 15/3 | FET=1.76; p=0.69 | |
| Age of Onset (years) | 16.5 (12) | 16.2 (15) | t(35)=0.0694; p=0.95 | |
| **Duration (years)** | **18.1 (14)** | **17.7 (13)** | **t(35)=0.10; p=0.92** | |
| # Current ASMs | 2.42 (0.96) | 2.39 (0.98) | t(35)=0.101; p=0.92 | |
| MTS (Y/N) | 10/9 | 7/11 | FET=0.582; p=0.51 | |
| Seizure Frequency**^#^** | 5.89 (6.9) | 3.62 (3) | t(34)=1.25; p=0.22 | |
| Engel Outcome (I/II+) | **15/4** | **11/6** | **FET; p=0.46** | |
| TLE: temporal lobe epilepsy; F: females; M: males; L: left; R: right; T: typical; A: a-typical; Y: yes; N: no; **WTAR: Weschlers Test of Adult Reading**  Standard deviations are presented inside the parentheses.  **^#^Seizure frequency includes both focal seizures as well as tonic clonic seizures.** | | | |  |

**Supplementary Table 2.** Correlation results of control tracts and ROIs.

|  | Pre- surgical BNT | Pre- surgical ANT |  | L-TLE  Post- surgical BNT | L-TLE  Post- surgical ANT |  | R-TLE  Post- surgical BNT | R-TLE  Post- surgical ANT |
| --- | --- | --- | --- | --- | --- | --- | --- | --- |
| L-Amygdala **(Volume)** | -0.004 | -0.02 |  | -0.17 | -0.15 |  | -0.16 | 0.02 |
| R-Amygdala (**Volume)** | -0.15 | -0.09 |  | 0.09 | 0.13 |  | -0.13 | -0.06 |
| L-CST **(FA)** | -0.03 | 0.07 |  | -0.05 | -0.39 |  | 0.23 | -0.11 |
| R-CST **(FA)** | -0.07 | -0.02 |  | 0.22 | -0.09 |  | 0.18 | 0.18 |
| L-Entorhinal **(FA)** | 0.08 | 0.11 |  | 0.06 | -0.19 |  | 0.43 | -0.18 |
| R-Entorhinal **(FA)** | -0.04 | 0.10 |  | 0.37 | 0.39 |  | -0.05 | -0.3 |
| *p<.05; **p<.01; **Significant effects that survived FDR correction are bolded.**  TLE: temporal lobe epilepsy; BNT: boston naming test; ANT: auditory naming test; CST: cortico-spinal tract; L: left; R: right | | | | | | | | |

**Supplementary Table 3.** Pre-surgical correlation results for typical language dominance patients only.

|  | Pre- surgical BNT | Pre- surgical ANT |  | LTLE  Post- surgical BNT | LTLE  Post- surgical ANT |  | RTLE  Post- surgical BNT | RTLE  Post- surgical ANT |
| --- | --- | --- | --- | --- | --- | --- | --- | --- |
| Pre-surgical Score | - | - |  | -0.56 | -0.27 |  | -0.028 | -0.55* |
| Education | **0.4**** | **0.36**** |  | 0.093 | -0.039 |  | -0.58* | -0.11 |
| Age 0f Onset | **0.41**** | 0.29* |  | -0.071 | -0.13 |  | -0.22 | -0.25 |
| # ASMs | 0.17 | 0.021 |  | -0.45 | -0.42 |  | 0.074 | 0.22 |
| L-Hippocampus **(Volume)** | 0.12 | 0.12 |  | -0.36 | -0.13 |  | -0.2 | -0.057 |
| R-Hippocampus **(Volume)** | 0.0051 | 0.078 |  | -0.041 | -0.014 |  | -0.34 | -0.36 |
| L-ILF **(FA)** | **0.26**** | **0.34**** |  | 0.36 | 0.28 |  | 0.65* | 0.19 |
| R-ILF **(FA)** | **0.25**** | **0.35**** |  | 0.23 | -0.2 |  | 0.52 | -0.088 |
| L-IFOF **(FA)** | **0.29**** | 0.24* |  | 0.13 | 0.51 |  | 0.25 | -0.45 |
| R-IFOF **(FA)** | **0.36**** | **0.33**** |  | 0.42 | -0.22 |  | 0.51 | -0.12 |
| L-Fusiform **(FA)** | **0.27**** | **0.3**** |  | 0.24 | -0.28 |  | 0.4 | -0.083 |
| R.-Fusiform **(FA)** | 0.17 | 0.15 |  | **0.85**** | 0.096 |  | 0.14 | -0.3 |
| *p<.05; **p<.01; **Significant effects that survived FDR correction are bolded.**  TLE: temporal lobe epilepsy; BNT: boston naming test; ANT: auditory naming test; ASMs: anti-seizure medications; ILF: inferior longitudinal fasciculus; IFOF: inferior longitudinal fasciculus; L: left; R: right | | | | | | | | |

**
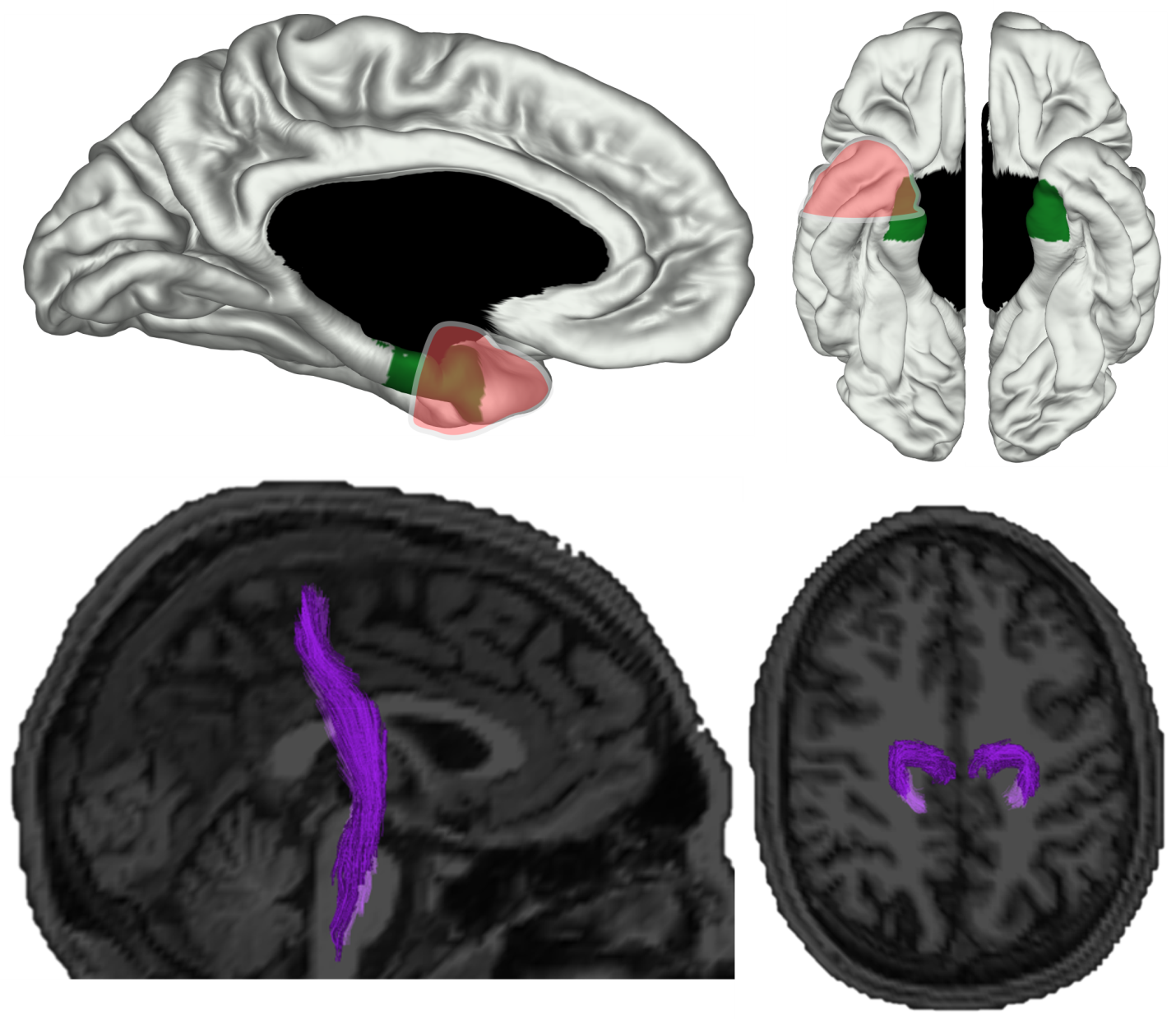
**

**Supplementary Figure 1. Illustration of control regions included in additonal analysis.** Top row) Sagittal and ventral view of the SWM ROI of the entorhinal region (green) derived from the Desikan-Killiany Atlas and an illustration of the approximate extent of an ATL performed on the left hemisphere. Bottom row) Sagittal and coronal views of the cortico-spinal tract (purple) derived from AtlasTrack and projected onto a T1-weighted image for a single individual.

**
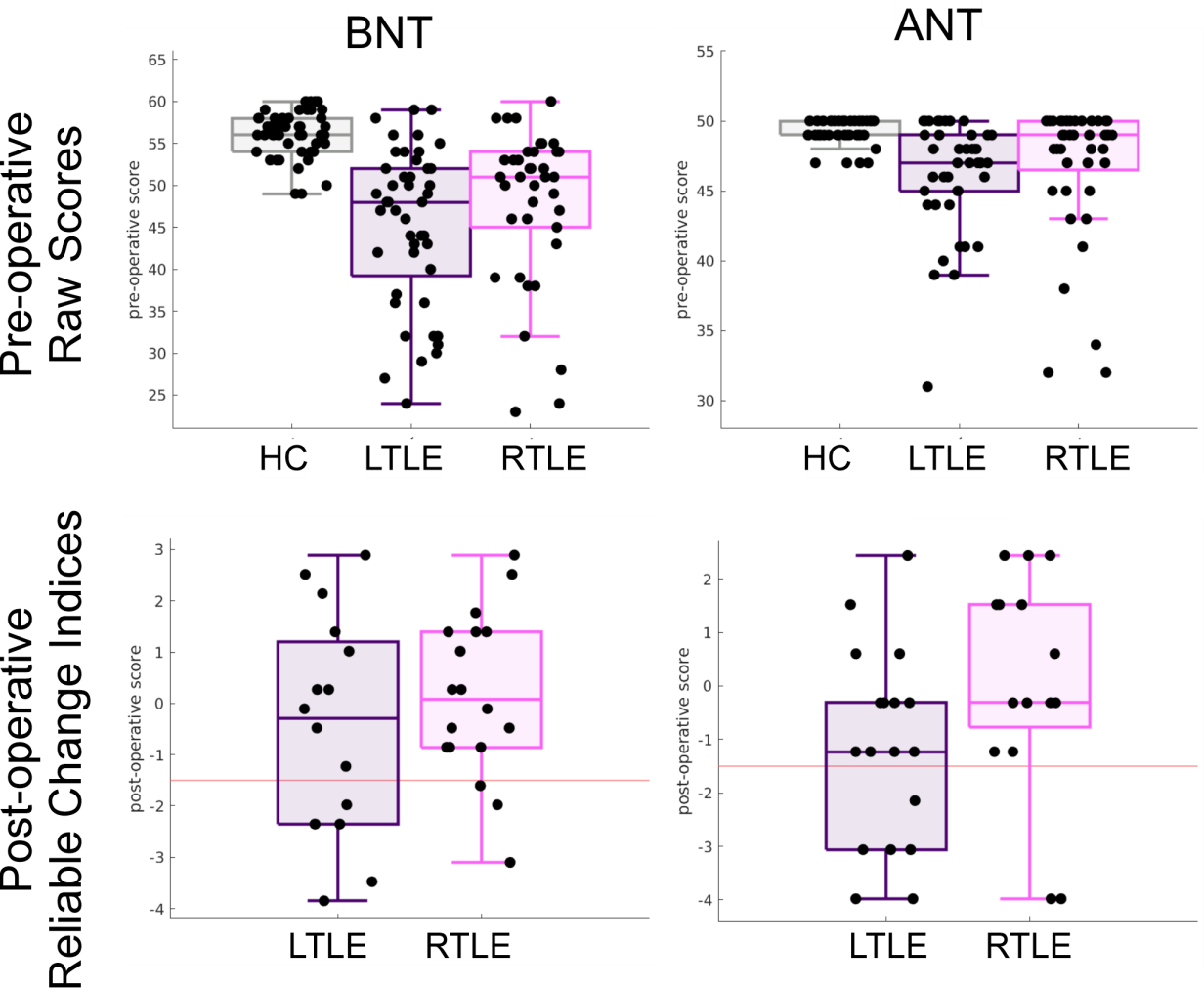
**

**Supplementary Figure 2. Naming performance across patient groups.** Top row**:** Pre-surgical scores for healthy controls (HC; grey), left temporal lobe epilepsy (purple, L-TLE), and right temporal lobe epilepsy (pink; R-TLE). Bottom row: Post-surgical reliable change indices for left temporal lobe epilepsy (purple, L-TLE) and left temporal lobe epilepsy (pink; R-TLE). Red line indicates -1.5 standard deviation cutoff for impairment classification.
